# Food Bioactives: Impact on Brain and Cardiometabolic Health—Findings from In Vitro to Human Studies

**DOI:** 10.3390/foods10051045

**Published:** 2021-05-11

**Authors:** Nenad Naumovski, Domenico Sergi

**Affiliations:** 1 Discipline of Nutrition and Dietetics, Faculty of Health, University of Canberra, Canberra, ACT 2601, Australia; 2 Functional Foods and Nutrition Research (FFNR) Laboratory, University of Canberra, Bruce, ACT 2617, Australia; 3 Discipline of Nutrition-Dietetics, School of Health Science and Education, Harokopio University, 17671 Athens, Greece; 4 Molecular Nutrition, Department of Medical Biology, Université du Québec à Trois-Rivières, Trois-Rivières, QC G8Z 4M3, Canada; Domenico.sergi2@uqtr.ca or; 5 Adelaide Medical School, The University of Adelaide, Adelaide, SA 5000, Australia

Modern society is currently (and probably more than ever) immersed in the changing concept of food, seeking the beneficial functions of foods rather than only as a mean to quench hunger and support basic nutritional needs. In this context, we are facing a change in the expectations that consumers have from food items, accompanied by an increased attention towards food bioactive derivatives with health boosting properties. These emerging perceptions of food as a key discriminant in human health are fueled by the already strong evidence linking unhealthy dietary patterns with the onset and progression of several chronic diseases, ranging from type 2 diabetes mellitus (T2DM) to cancer and neurodegenerative diseases. On the contrary, functional foods and their bioactive components may represent a nutritional cornerstone to improve the quality of diet and ameliorate or prevent (in some cases) nutrition-related diseases. Bioactives are unlike pharmaceuticals (compounds used to alleviate symptoms and cure disease). Nevertheless, the latest findings indicate that the clear gap between the two products (bioactives and pharmaceuticals) is becoming narrower and in some cases, they are becoming interchangeable. 

In agreement with the aforementioned considerations, the interest of the general population with respect to functional foods containing bioactive molecules is in constant expansion, which provides an impetus for research in this field. Indeed, several studies, including in vitro investigations, clinical trials and observational studies related to food and dietary patterns have already identified, proposed and in some cases, challenged the mechanisms of action of food bioactive derivatives. Therefore, the main aim of this Special Issue was to provide an opportunity to bring together high-quality manuscripts that showcase the current knowledge in relation to food bioactives and their impact on brain and cardiometabolic health. 

The article by Deutch et al. (2019) is a comprehensive work identifying the compositional properties of licorice and the potential impacts on blood pressure and the cardiovascular system [1]. Licorice is the root of the legume *Glycyrrhiza glabra* that is commonly grown in warm climatic areas such as the Middle East, Asia and southern parts of Europe. For several millennia, this root was used in traditional medicines of many countries as an ailment for a number of different diseases and heath conditions, such as gastrointestinal symptoms and respiratory diseases. Nowadays, a broad-spectrum of health-related properties have been ascribed to licorice, including immunostimulatory effects; anti-ulcer, anti-cancer, anti-viral and anti-microbial effects; in addition to the protection of the nervous and cardiovascular systems. Although licorice consists of over 300 potential bioactive compounds, the authors report that the health effects elicited by licorice mainly rely on the bioactive glycyrrhizin. This molecule is a prodrug that is converted into 3β-monoglucuronyl-18β-glycerrhetinic acid (3MGA) and 18β-glycerrhetinic acid (GA) in the small intestine. Despite both compounds having been associated with a variety of potential health benefits, 3MGA and GA can also inhibit the hydroxysteroid dehydrogenase II enzyme, which is responsible for oxidizing cortisol into cortisone. Therefore, high licorice consumption can also potentially promote hypernatremia, hypokalemia and fluid volume retention. Furthermore, the authors report on the findings from a relatively recent meta-analysis where the increased intake of licorice was associated with significant increases in systolic (5.45 mmHg) and diastolic (3.19 mmHg) blood pressures (BPs). The authors also propose caution against the consumption of large quantities of licorice as some negative health effects may occur. 

The incidence and occurrence of neurodegenerative diseases is on a constant increase worldwide, with Alzheimer’s Disease (AD) and Parkinson’s Disease (PD) being the most prevalent. A pivotal pathophysiological aspect of these diseases is the progressive neuronal loss that can be triggered by oxidative stress, mitochondrial dysfunction and neuroinflammation. In this regard, strategies aimed at counteracting the damaging effects of oxidative stress and neuroinflammation are considered as promising avenues to prevent neurodegeneration. In this context, bioactive molecules may play a role in counteracting the pathophysiological mechanisms linked with neurodegeneration. Of these, ε-viniferin (resveratrol dimer), as reported in the paper by Sergi et al., has shown to share similar effects with resveratrol in relation to neuroprotection in animal models of AD and Huntington’s Disease [2]. Nevertheless, the effects of ε-viniferin on oxidative stress and inflammation-induced injury in dopaminegeric neurons remain relatively unexplored. In this study, the authors reported the neuroprotective potential of ε-viniferin in nerve growth factor (NGF)-differentiated PC12 cells, an in vitro dopaminergic model of Parkinson’s disease (PD), and assessed the potential anti-inflammatory properties of this nutraceutical in a N9 microglia-neuronal PC12 cell co-culture system. The cells were pretreated with ε-viniferin, resveratrol and their mixtures before the administration of 6-hydroxydompamine (6-OHDA) that is recognized for inducing PD-like symptoms in animal models. In addition, the authors also investigated the effects of these stilbenes on the potential reduction in lipopolysaccharide-induced inflammation. The findings indicated that ε-viniferin alone or in combination with resveratrol protects the neuronal dopaminergic PC12 cells from 6-OHDA-induced cytotoxicity and apoptosis as well as the neuronal cytotoxicity triggered by microglial activation. 

L-Theanine (L-THE) is the most abundant non-proteinogenic amino acid found in green tea. Its consumption has been proposed to be associated with stress-reducing effects and antihypertensive properties as well as improvements in cognitive functioning. In consideration of this, and given the recent commercial availability of relatively pure L-THE, there is a strong potential for the development of functional food products that contain this amino acid as a bioactive ingredient. A study by Williams et al. (2020) investigated the physiological responses, including heart rate (HR), heart rate variability (HRV) and BP in healthy males (*n* = 11), following the acute ingestion of a functional food products (mango sorbet) containing 200 mg of pure L-THE [3]. In this double blind, placebo-controlled cross-over trial, the participants were required to consume the test food or placebo mango sorbet (color and flavor matched) after which their physiological responses were continuously monitored over a period of 90 min. The study reported no significant differences between the intervention and placebo or within the individual groups (all *p*’s > 0.05). Furthermore, the results of this study indicate that there was also no parasympathetic response following L-THE intake (determined via the HR response). The authors have proposed that these findings could potentially be due to the interaction between L-THE and the food matrix it was embedded in, which may have affected L-THE bioavailability. In addition, the authors have acknowledged the limitations of the study, such as the sample size (considering this being the pilot trial) and the selection of healthy participants. Nevertheless, this study highlights the importance of the careful selection of the food matrix composition in the development of future functional foods containing L-THE as an active ingredient, particularly considering that the matrix itself may affect the bioavailability of bioactive molecules.

The urbanization of sub-Saharan Africa is associated with a dietary shift towards the overconsumption of energy-dense foods, which in turn significantly contributes to the overall increase in cardiovascular disease, obesity and type 2 diabetes observed in this geographical area. A study by Zec et al. (2019) investigated the South African cohort of the Prospective Urban Rural Epidemiology (PURE) study, an international study investigating the health complications associated with urbanization in *low-*, *middle-* and *high-*income countries [4]. In this study, the authors examined data from 300 adults (older than 30 years of age) on the occurrence of hypertension and long-chain polyunsaturated fatty acids (PUFA) status. Data were analyzed from three consecutive examinations (2005, 2010 and 2015). The results revealed that the ten-year hypertension incidence significantly increased among rural participants (+20%, *p* = 0.001) while there was no significant change in urban participants (*p* = 0.253). Moreover, regardless of urbanization, n-6 PUFA status increased while the eicosapentanoic acid (EPA) status decreased over the 10-year period. Furthermore, authors reported an increase in BP (+2.92 systolic and +1.94 mmHg diastolic) and 1.46 higher odds of being hypertensive in the participants in the highest EPA tertile. In black South Africans included in this study sample, individual plasma n-6 PUFA were inversely associated with BP while EPA was associated with increases in BP leading to hypertension. Nonetheless, these findings should be interpreted with caution, especially in consideration of the well-documented positive effects of omega-3 fatty acids, such as EPA, on cardiovascular health.

The study by Leal-Martinez et al. (2020) is an exploratory randomized controlled clinical trial investigating the effects of a nutritional support system for improving motor function in children living with cerebral palsy (CP) in Mexico [5]. CP is one of the most common disabilities in childhood and changes to gross motor function is one of the main characteristics associated with this disease that can contribute to malnutrition in patients with CP. Furthermore, parasitosis is very common in this population sample and can contribute to the impairment of nutrient absorption. In this study, children with CP (*n* = 30) were divided in the three groups (*n* = 10 each): follow-up (monitoring of the diet only), control (dewormed and received nutritional therapy recommended by WHO) and intervention group (dewormed and received nutritional support system (diet and supplements)). All participants received Bobath physical therapy. The supplemental composition included a combination of amino acids (glutamine and arginine), vitamins (folic acid, cholecalciferol, ascorbic acid, nicotinic acid), minerals (zinc and selenium), spirulina, vegetable-based protein and n-3 PUFA. In addition, the participants also consumed probiotics (200 mg of *Saccharomyces Bouladii*) every 12 h for three days in the basal period and at week 7 for correcting malabsorption. The overall findings of the study were that a nutritional intervention consisting of diet and combination of supplements resulted in an improvement in gross motor function and promoted increases in walking ability in children living with CP.

In summary, the field of food bioactives is exceptionally diverse and it is a rapidly growing area of research and development. This is also fueled by the availability of novel analytical techniques, experimental models of human disease, and by an increase in consumer demand, seeking food products supported by solid health claims. Indeed, in order to meet these expectations, there is an ever-increasing need to translate the discoveries related to the health-promoting effects of food bioactives from the bench to the clinic, particularly in relation to brain and cardiometabolic health which represent areas of research with the greatest potential impact of human health. The findings reported in the studies published as part of this Special Issue further confirm the potential beneficial role of food bioactive derivatives and pave the way for future investigations aimed at further dissecting the impact of food bioactive molecules on human health. Nevertheless, we must not overlook the fact that food bioactives should not be considered as a panacea in the battle against human chronic diseases, as their health promoting effects cannot be sustained or even occur in the absence of a healthy lifestyle characterized by adherence to healthy dietary patterns and physical activity. We (the co-editors of this Special Issue) are thankful to the authors and reviewers who have contributed to this issue by sharing their knowledge, findings and time. 

## References

[1] Deutch M.R., Grimm D., Wehland M., Infanger M., Kruger M. (2019). Bioactive Candy: Effects of Licorice on the Cardiovascular System. Foods.

[2] Sergi D., Gelinas A., Beaulieu J., Renaud J., Tardif-Pellerin E., Guillard J., Martinoli M.G. (2021). Anti-Apoptotic and Anti-Inflammatory Role of Trans epsilon-Viniferin in a Neuron-Glia Co-Culture Cellular Model of Parkinson’s Disease. Foods.

[3] Williams J., McKune A.J., Georgousopoulou E.N., Kellett J., D’Cunha N.M., Sergi D., Mellor D., Naumovski N. (2020). The Effect of L-Theanine Incorporated in a Functional Food Product (Mango Sorbet) on Physiological Responses in Healthy Males: A Pilot Randomised Controlled Trial. Foods.

[4] Zec M.M., Schutte A.E., Ricci C., Baumgartner J., Kruger I.M., Smuts C.M. (2019). Long-Chain Polyunsaturated Fatty Acids Are Associated with Blood Pressure and Hypertension over 10-Years in Black South African Adults Undergoing Nutritional Transition. Foods.

[5] Leal-Martinez F., Franco D., Pena-Ruiz A., Castro-Silva F., Escudero-Espinosa A.A., Rolon-Lacarrier O.G., Lopez-Alarcon M., De Leon X., Linares-Eslava M., Ibarra A. (2020). Effect of a Nutritional Support System (Diet and Supplements) for Improving Gross Motor Function in Cerebral Palsy: An Exploratory Randomized Controlled Clinical Trial. Foods.

